# Supplementary material for: Neuroanatomy accounts for age-related changes in risk preferences
Source: Nat Commun. 2016 Dec 13;7:13822. doi: 10.1038/ncomms13822 (PMC5159889; doi:10.1038/ncomms13822)
Supplement: Supplementary Information — Supplementary Methods and Supplementary References. [file ncomms13822-s1.pdf]

Supplementary Methods

We included the ambiguous trials in analyses analogous to the ones presented in the main text and modeled the expected utility (EU) of each option using the same function employed in References 1 and 2. This function[3] takes into account the effect of ambiguity on the perceived probability, and both an ambiguity attitude parameter (β) and a risk attitude parameter (α) can be estimated:

$$EU(v, p, A) = [p - \beta \frac{A}{2}] \times v^{\alpha}$$

where v = value (amount), p = probability, A = degree of ambiguity, β = the ambiguity attitude parameter, and α = the risk preference parameter. We allowed β, the ambiguity attitude parameter, to vary during the estimation procedure as a function of age (Model 1), as a function of age and rPPC gray matter volume (Model 2), and as a function of age, rPPC gray matter volume, and global gray matter volume (Model 3). We found no relationship between ambiguity aversion and any of these predictors, in any of the models (all p values > 0.2) In a fourth model, we controlled for the relationship between risk attitude and rPPC gray matter volume by also allowing the risk attitude parameter to vary as a function of rPPC gray matter volume during the estimation procedure. Again, we found no relationship between ambiguity aversion and any of the predictors (age, rPPC gray matter volume, global gray matter volume; all p values > 0.2), and as expected, we found a significant positive relationship between risk attitude and rPPC gray matter volume (Z-test, n = 3,077, SEs clustered on 52 participants, z = 3.56, p = 0.0004).

Supplementary References

1 Tymula, A., Rosenberg Belmaker, L. A., Ruderman, L., Glimcher, P. W. & Levy, I. Like cognitive function, decision making across the life span shows profound age-related changes. *Proc Natl Acad Sci U S A* **110**, 17143-17148, doi:10.1073/pnas.1309909110 (2013).

2 Gilaie-Dotan, S. *et al.* Neuroanatomy predicts individual risk attitudes. *J Neurosci* **34**, 12394-12401, doi:10.1523/JNEUROSCI.1600-14.2014 (2014).

3 Gilboa I & Schmeidler, D. Maxmin expected utility with non-unique prior. *J Math Econ* **18**, 141–153 (1989).
